# A standardizable human-based psoriasis skin model for drug development

**DOI:** 10.3389/fmed.2025.1539484

**Published:** 2025-06-25

**Authors:** Hanna Glasebach, Steffen Rupp, Anke Burger-Kentischer

**Affiliations:** Fraunhofer Institute for Interfacial Engineering and Biotechnology IGB, Stuttgart, Germany

**Keywords:** in vitro skin model, psoriasis, T cells, STAT3, non-animal method

## Abstract

Psoriasis is a chronic inflammatory autoimmune disease with a prevalence of 2–3% in the western population. For the development of urgently needed new therapeutic options, standardizable and human-relevant disease models are essential. The transcription factor signal transducer and activator of transcription 3 (STAT3) plays a key role in the pro-inflammatory response of psoriatic keratinocytes. In this study, we established STAT3 overexpressing human keratinocytes and combined the cells with our epidermis and immune cell supplemented full-thickness skin model to generate a novel in vitro psoriasis model. In the full-thickness skin models STAT3 overexpression by its own was sufficient to induce enhanced expression of the psoriasis marker S100A7. Both epidermal and full-thickness skin models set up from STAT3 overexpressing keratinocytes showed a stronger psoriasis-like phenotype upon pro-inflammatory stimuli such as treatment with cytokines or integration of T cells compared to skin models set-up from the wild-type keratinocyte cell line. Thus, the STAT3 overexpression in keratinocytes induced a pre-psoriatic like phenotype in untreated skin models and enhanced the sensitivity of the skin models to psoriatic stimuli. This reflects the genetic susceptibility in psoriasis patients, which is responsible for the differences between non-lesional skin in patients and healthy skin. The novel STAT3 overexpressing in vitro skin model mimics many psoriatic hallmarks, is based on well-established immortalized primary keratinocytes and highly reproducible. Therefore, we expect it to be a well-suited psoriasis model for drug screening and validation approaches in a preclinical setting.

## 1 Introduction

Psoriasis is a chronic inflammatory autoimmune disease of the skin with a prevalence of 2–3% in the western population (1). It is characterized by a dysregulated cross talk between keratinocytes and immune cells (2, 3). A feedforward loop consisting of dendritic cells, T helper (Th) cells (e.g., Th17, Th22) and keratinocytes that respond to immune cell activation with further secretion of cytokines, chemokines and antimicrobial peptides drives the disease pathology (4). The inflammatory environment marked by elevated levels of cytokines leads to exceeding proliferation and abnormal differentiation of keratinocytes which results in histopathological hallmarks such as acanthosis and parakeratosis (5). Psoriasis is a complex multifactorial disease. One of the factors is genetic predisposition. More than 60 risk loci have been identified in psoriasis patients, including signal transducer and activator of transcription 3 (STAT3) (6, 7).

The transcription factor STAT3 is part of the JAK/STAT signaling pathways that is activated in response to cytokines or growth factors (8). STAT3 regulates a wide range of proliferation and inflammation associated genes (9). In keratinocytes the STAT3 signaling pathway is involved in wound healing, epidermal differentiation and expression of chemokines and antimicrobial peptides (10, 11). Nearly 20 years ago, Sano et al. (12) described the relevance of STAT3 in the pathology of psoriasis: They reported high levels of activated STAT3 in psoriatic skin (12). In the following years, many other studies confirmed STAT3 as a key player for disease pathogenesis (9). STAT3 signaling pathways play a key role in psoriasis associated immune cells (especially Th17 cells) as well as in psoriatic keratinocytes. STAT3 is activated in keratinocytes by a wide range of psoriasis-associated cytokines [e.g., interleukin (IL) 6, IL-22, IL-23 and IL-17] and mediates psoriatic hallmarks such as hyperplasia, increased proliferation and expression of the autoantigen keratin 17 (12). Recently, an endogenous inhibitor of STAT3, PIAS3, was found to be downregulated in psoriatic lesions (13), indicating a pathological hyperactivation of STAT3 in psoriatic keratinocytes. Thus, STAT3 is considered to be a potential target for the therapy of psoriasis and STAT3 inhibitors are investigated as a treatment option. Various experiments with mice and a first non-randomized clinical trial showed that blocking of STAT3 with a small molecule, STA-21, reduced or prevented the psoriatic phenotype (14). Drugs targeting other branches of the JAK/STAT pathway are currently investigated in several clinical trials with psoriasis patients (15).

The development of new treatment options requires an understanding of pathogenesis and well-characterized and readily available reproducible preclinical models to determine the safety and efficacy of new drugs. There is a wide variety of different preclinical psoriasiform mouse models that have significantly contributed to our knowledge of the disease, e.g., they provided insights on the pathologic mechanism, the role of certain risk genes and the connection of psoriasis with comorbidities. Further, they were successfully used for the validation of new targets and psoriasis treatments. There are spontaneous, imiquimod or cytokine induced, xenotransplanted or genetically modified mouse models that each reflect different aspects of the pathogenesis (16–18). One prominent genetically engineered mouse model is based on expression of a constitutive active form of STAT3 in keratinocytes (K5.STAT3C mice) (12). This leads to the development of psoriasis-like lesions in newborn mice, which were manifested in high Ki-67 expression and impaired keratinocyte differentiation. Thus, in K5.STAT3C mice, the abundance and hyperactivation of STAT3 in psoriasis was successfully used to recapitulate some psoriatic characteristics in murine models, confirming STAT3 as a possible target for psoriasis treatment. However, transferability of results from mice to humans is limited due to major species-specific differences in the skin anatomy, immune cell populations and innate immunity [reviewed in detail in (17, 19)]. γδ T cells that mediate psoriasis-like phenotypes in many mouse models but do not play a role in human psoriasis are just one example why results from animal models have to be evaluated with care (20). Alternatives to mice are in vitro skin models based on human keratinocytes and fibroblasts which are already used in the cosmetic and personal care industry for safety testing. Still, a current research focus remains the recapitulation of psoriatic characteristics with *in vitro* skin equivalents. So far, there are several different approaches described in the literature: (1) by use of diseased cells or tissue from psoriasis patients (21–24), (2) by addition of cytokines (25–31) and (3) by integration of immune cells to epidermal and full thickness skin models (23, 29, 32, 33). A recent review summarizes 45 publications from 2011 to 2021 on the current in vivo, in vitro and ex vivo preclinical models for psoriasis and discusses their advantages and limitations (34). Although psoriasis is a disease with genetic predisposition, in contrast to numerous published genetic engineered mouse models no in vitro models with defined genetic modifications have been reported. Ex vivo models from patients could compensate for this limitation, however, the exact nature of the genetic predisposition in general is not known and their availability is limited. While, even though, STAT3 activation was successfully used to mimic psoriatic conditions in murine models, this was so far not transferred on human in vitro models.

To establish a human-based preclinical in vitro model for treatment development in psoriasis, we sought to generate an immunocompetent 3D model with a defined genetic modification predisposing to psoriasis, namely STAT3 overexpression. With the model, we can combine different cell types and their genetic modifications in a modular manner to focus on individual targets and increase the levels of complexity in a defined and reproducible way. In previous work, we already developed an immunocompetent 3D in vitro model for *C. albicans* infections, which contains an epidermis, a dermal part with fibroblasts and an immune cell layer (35). The interaction between the different cell types in the model was successfully demonstrated (36). In this study, we adapted the immunocompetent skin model to a psoriatic phenotype by (1) developing a STAT3 overexpressing human keratinocyte cell line and (2) integrating psoriasis-relevant CD4^+^ T cells. Thereby we established to our knowledge for the first time a 3D in vitro psoriasis model with a defined psoriasis-predisposing genetic modification. This psoriasis model was extensively characterized confirming a psoriatic phenotype for all parameters investigated. Our results furthermore indicate a significant impact of the activation state of immune cells added to the psoriasis models, showing the advantages of the modular concept.

## 2 Materials and methods

### 2.1 Cell culture

The human keratinocyte cell line Ker-CT (ATCC, CRL-4048) was cultivated in DermaLife K keratinocyte growth medium (CellSystems, LM-0027) with 1% penicilin-streptomycin. Human primary fibroblasts were cultivated in Dulbecco’s modified Eagles’s medium (DMEM) with 10% fetale calf serum (FCS), 2 mM L-glutamine and 1% penicillin–streptomycin. Basic culture medium for human T cells was Roswell-Park-Memorial-Institute (RPMI) 1640 medium with 10% FCS, 2 mM L-glutamine and 1% penicillin-streptomycin.

### 2.2 Establishment of the monoclonal STAT3 overexpressing keratinocyte cell line

A DNA sequence coding for the human STAT3 (NCBI reference number NP_001356441.1) including a C-terminal enhanced Green Fluorescent Protein (eGFP) tag was synthesized by GeneArt (Thermo Fisher Scientific Inc.) and subsequently integrated in the plasmid pReceiver-blast via restriction enzyme cloning with XhoI and BstBI. pReceiver-blast was created by change of the neomycin to a blasticidin resistance gene in the plasmid pReceiver-M02 (rzbd). The cloned plasmid was stably transfected into Ker-CT keratinocytes by nucleofection with the Amaxa™ cell line nucleofector™ 2b device [program T018, human keratinocyte nucleofector™ kit (Lonza, VPD-1002)]. Transfected Ker-CT were cultured in DermaLife K keratinocyte growth medium (CellSystems, LM-0027) supplemented with 1.5 μg/mL blasticidin for 14 days. Monoclonal cell lines were established by single cell sorting (Sony SH800S). The different cell clones were analyzed for STAT3-eGFP expression with a western blot for STAT3 and immunofluorescence microscopy for eGFP in 2D culture. Further, the cell clones were differentiated in 3D epidermis models and analyzed histological and immunohistochemical for stratification and STAT3-eGFP expression (see section 2.5 and 2.8). One STAT3 overexpressing cell clone was chosen for the further experiments, hereafter named Ker-CT_STAT3.

### 2.3 Stimulation of keratinocytes with cytokines and western blot analysis

Ker-CT and Ker-CT_STAT3 (80,000 cells per well) were seeded in 6-well plates in keratinocyte growth medium. On day 3 after seeding, cells were stimulated with cytokines (20 ng/mL IL-22, 20 ng/mL IL-17, 20 ng/mL IFN-γ). After 48 h, the cell monolayer was washed with PBS and lysed in 200 μL RIPA buffer per well on ice for 10 min. The protein concentration in the cell lysates was determined by a BCA assay. The denatured protein (10 μg per sample) was separated by SDS gel electrophoresis in a NuPage® Novex® 4–12% Bis-Tris protein gradient gel (Invitrogen). Separated proteins were transferred on a nitrocellulose membrane by semi-dry blotting at 20 V for 7 min. The membrane was blocked in 5% milk powder in TBS-T and incubated with the primary antibody in 5% milk powder/TBS-T or 5% BSA/TBS-T overnight at 4°C. The following primary antibodies were used: anti-S100A7 (novus biologicals, cat no NB100-56559, 1:500), anti-phospho-STAT3 (abcam, cat no ab76315, 1:1000), anti-STAT3 (abcam, cat no ab119352, 1:1000) and anti-GFP (abcam, cat no ab290, 1:1000). *β*-actin (cell signaling technology, cat no 4970S, 1:5000) was used as loading control. The next day, the secondary horseradish peroxidase (HRP) coupled antibody (anti-rabbit from Sigma-Aldrich, cat no A0545 and anti-mouse from Jackson Immuno Research, cat no 115-035-068) was applied in 5% milk powder/TBS-T for 1 h at room temperature and afterwards the membrane was developed with Pierce™ ECL western blot substrate.

### 2.4 T cell isolation and activation

Peripheral blood mononuclear cells (PBMCs) were isolated from blood of healthy donors by density centrifugation. The CD4^+^ cells were purified from PBMCs by negative selection with CD4^+^ T cell isolation kit (miltenyi biotec, cat no 130–096-533) according to the manufacturer’s instructions. Sorted CD4^+^ T cells were ≥95% pure. T cells were transferred in a non-treated cell culture flask at a density of 1-2 × 10^5^ cells/cm^2^ and cultured in RPMI 1640 in the presence of Dynabeads™ CD3/CD28 human T activator (1 bead/4 cells) and 30 U/mL IL-2 for up to 1 week.

### 2.5 Generation of epidermal equivalents

Keratinocytes (0.15×10^6^ cells/insert) were seeded in 0.4 μm Nunc trans-well cell culture inserts and cultured submerse in DermaLife K keratinocyte submerse medium (DermaLife K keratinocyte growth medium supplemented with 1.86 mM CaCl_2_). After 3 days, the inserts were transferred in a 6-well plate and 2 mL DermaLife K keratinocyte differentiation medium (DermaLife K keratinocyte growth medium supplemented with 1.86 mM CaCl_2_ and 50 μg/mL ascorbic acid) was added. The cells were fed from below and the amount of differentiation medium was adapted in a way that the surface of the keratinocyte layer remained constantly dry. The medium was changed every 2–3 days. At day 5 of the airlift culture, cytokines (20 ng/mL IL-22, 20 ng/mL IL-17) were added to the cell culture medium. At day 11, the constructs were subjected to histological and immunhistochemical staining.

### 2.6 Generation of immune cell supplemented full-thickness human skin models

The 3D skin construct consisted of a keratinocyte layer seeded on top of a collagen matrix with fibroblasts (35). In the immunocompetent system, a further collagen matrix, containing the immune cells, was added below the dermal compartment. Fibroblasts (50,000 cells/insert) resuspended in 500 μL rat tail collagen type I/gel neutralization solution [ratio 2:1, final collagen concentration 4 mg/mL (35)] were deposited in 8 μm BRAND trans-well cell culture inserts. The collagen gel was physically cross-linked for 30 min at 37°C, 5% CO_2_ before the inserts were placed submerse in complete DMEM. After incubation overnight at 37°C, the keratinocytes (150,000 cells/insert) were seeded in 100 μL DermaLife K keratinocyte growth medium on top of the collagen matrix. The constructs were cultured submerse in DermaLife K keratinocyte submerse medium for 3 days. At the beginning of the airlift phase, the inserts were transferred in a 6-well plate and 2–3 mL DermaLife K keratinocyte differentiation medium were added per well. Due to shrinkage of the collagen gel, the amount of differentiation medium was adapted to keep the surface of the keratinocyte layer dry. After 8 days of airlift culture, 300,000 immune cells were seeded in 200 μL collagen/gel neutralization solution (ratio 1:2) in a new 8 μm trans-well cell culture inserts. The collagen was physically cross-linked for 5–10 min at 37°C, 5% CO_2_ and the skin constructs were placed on top. The immunocompetent skin was cultivated for 3 days at the air-liquid interface in DermaLife K keratinocyte differentiation medium before analysis.

### 2.7 Analysis of metabolic activity in skin models

The metabolic activity was analyzed in the epidermis models with an alamarBlue™ cell viability assay (Thermo fisher scientific Inc., cat no DAL1025). Therefore, epidermis models were incubated in DermaLife K keratinocyte differentiation medium supplemented with alamarBlue™ reagent (1:10) for 3 h. The fluorescence in the supernatant was measured in triplicates: excitation wavelength 540 nm, emission wavelength 590 nm.

### 2.8 Histology and immunohistochemistry

Tissue sections were fixed in Bouins solution (Carl Roth GmbH) for 1 h, watered and embedded in paraffin with a routine program. Paraffin blocks were prepared, sliced and transferred to object carriers. Tissue slices were deparaffinized and stained with hematoxylin (Merck KGaA) and eosin (Sigma-Aldrich). Specific antigens were stained immunohistochemically. The tissue sections were deparaffinized, hydrated and masked epitopes were recovered by heat-induced antigen retrieval. The endogenous peroxidase was inhibited by incubating the tissue sections in 3% H_2_O_2_ for 10 min, followed by blocking with 5% BSA for 30 min and incubation with the primary antibody [anti-S100A7 (BioTechne Sales Corp., cat no NB100-56559, 1:500), anti-GFP (abcam, cat no ab290, 1:1000), anti-cytokeratin 16 (Thermo fisher scientific inc., cat no. MA5-13730, 1:500), anti-cytokeratin 10 (abcam, cat no ab9026, 1:1000), anti-IL-8 (Thermo fisher scientific inc., cat no PA5-79113, 1:350)] in Dako antibody diluent (Agilent Technologies, cat no S2022) at 4°C overnight. The biotin coupled secondary antibody (Biogenex, cat no LP000-ULE) in link diluent (1:100) was applied for 30 min, followed by incubation with the streptavidin linked peroxidase in label diluent (1:100) for 30 min. Between each step, the tissue sections were washed thrice in TBS-T. 3-Amino-9-Ethylcarbazole (AEC) chromogen diluted in peroxidase buffer (BioGenex, HK092-5K) was applied on the sections to visualize the protein expression. The tissue sections were counterstained with hematoxylin and mounted with Aquatex solution.

### 2.9 Quantification of immunohistochemical staining

Microscopic images of the stained tissue sections were aligned to reproduce a cross-section through the whole model. In the ImageJ software (37), the aligned microscopic images were converted to a HSB stack, the threshold was adjusted accordingly and the stained area in the entire model was measured. Data was normalized to the control condition and is represented as mean +/− standard deviation. For statistical analysis student’s *t*-tests were performed.

## 3 Results

### 3.1 Characterization of STAT3 overexpressing keratinocytes

**Figure 1:**
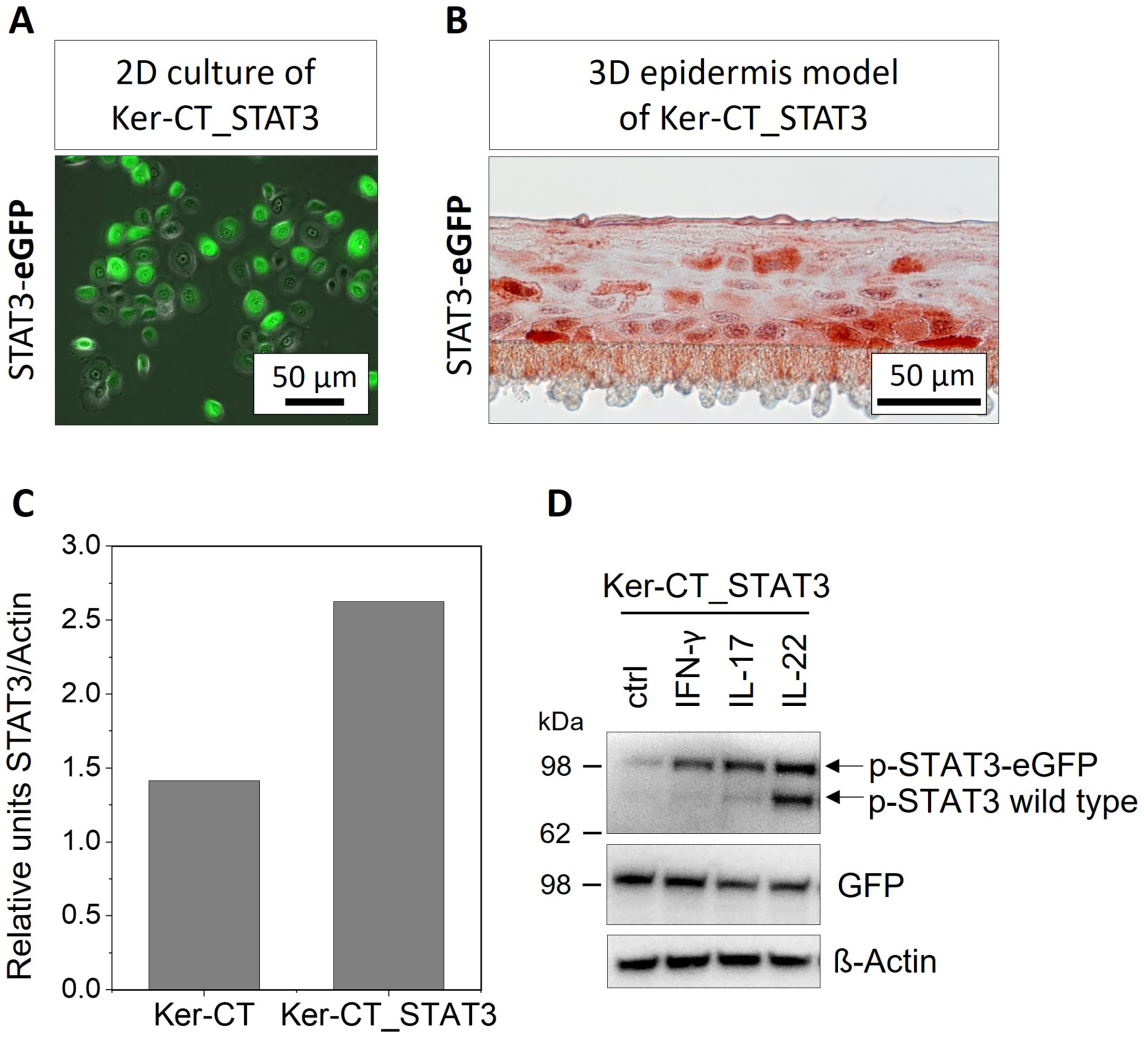
Characterization of Ker-CT_STAT3 keratinocytes. **(A)** Merged phase contrast and GFP fluorescence image of Ker-CT_STAT3 keratinocytes. STAT3-eGFP is expressed in all cells with varying intensity. **(B)** Epidermis equivalents were set up from Ker-CT_STAT3 and stained immunohistochemically for the exogenous eGFP tagged STAT3 with anti-GFP. STAT3-eGFP overexpressing keratinocytes are located predominantly in the *stratum basale* and *stratum spinosum*. **(C)** Western blot quantification of STAT3 expression (western blot not shown). The intensity of the STAT3 band (anti-STAT3) was normalized to the loading control actin. **(D)** Ker-CT_STAT3 were stimulated with IFN-y, IL-17 or IL-22 (each 20 ng/mL) for 48 h. After cell lysis, a western blot analysis for GFP and the phosphorylated STAT3 (p-STAT3, phosphorylation at Y705) was performed. Exogenous STAT3-GFP (upper band) and endogenous wild-type (wt) STAT3 (lower band) is phosphorylated in response to cytokine treatment.

We established a novel keratinocyte cell line that overexpressed constitutively an eGFP tagged STAT3 protein (Ker-CT_STAT3). The nuclear translocation and functionality of C-terminal eGFP tagged STAT3 was previously demonstrated (38). The eGFP tag enabled us to distinguish between the endogenous and the exogenous STAT3 in the cells. The morphology of the Ker-CT_STAT3 in 2D monolayer culture and the stratification in 3D epidermis models based on Ker-CT_STAT3 was not affected by the genetic modification (Figure 1A). In unstimulated Ker-CT_STAT3 the exogenous STAT3 was located throughout the cells as shown by immunofluorescence microscopy for eGFP. In the 3D epidermis models STAT3-eGFP expression was detected in all skin layers with immunohistochemically staining except for the *stratum corneum* (Figure 1C). Although Ker-CT_STAT3 are a monoclonal cell line, the STAT3 expression varied between cells of similar state of differentiation.

The novel established STAT3 overexpressing keratinocyte cell line was analyzed by western blotting for expression and phosphorylation of the exogenous STAT3 (Figure 1D). The expression level of STAT3 protein in Ker-CT_STAT3 cells was nearly twice as high if compared to the protein level in the base cell line Ker-CT (Figure 1C). Phosphorylation of STAT3 and STAT3-eGFP was analyzed by stimulation of the keratinocytes with different cytokines (IFN-γ, TNF-*α*, IL-17 and IL-22). Both the exogenous STAT3-eGFP and the endogenous STAT3 were phosphorylated at Y705 as indicated by western blotting using anti-phospho-STAT3 antibody (Figure 1D). The strongest phosphorylation of overall and exogenous STAT3, was achieved after treatment with IL-22. Interestingly, for all cytokine treatments, the phosphorylation of the exogenous STAT3-eGFP appeared stronger than the phosphorylation of the endogenous wild-type STAT3 (Figure 1D).

### 3.2 Stimulation of STAT3 overexpressing epidermis equivalents with psoriasis-associated cytokines

**Figure 2:**
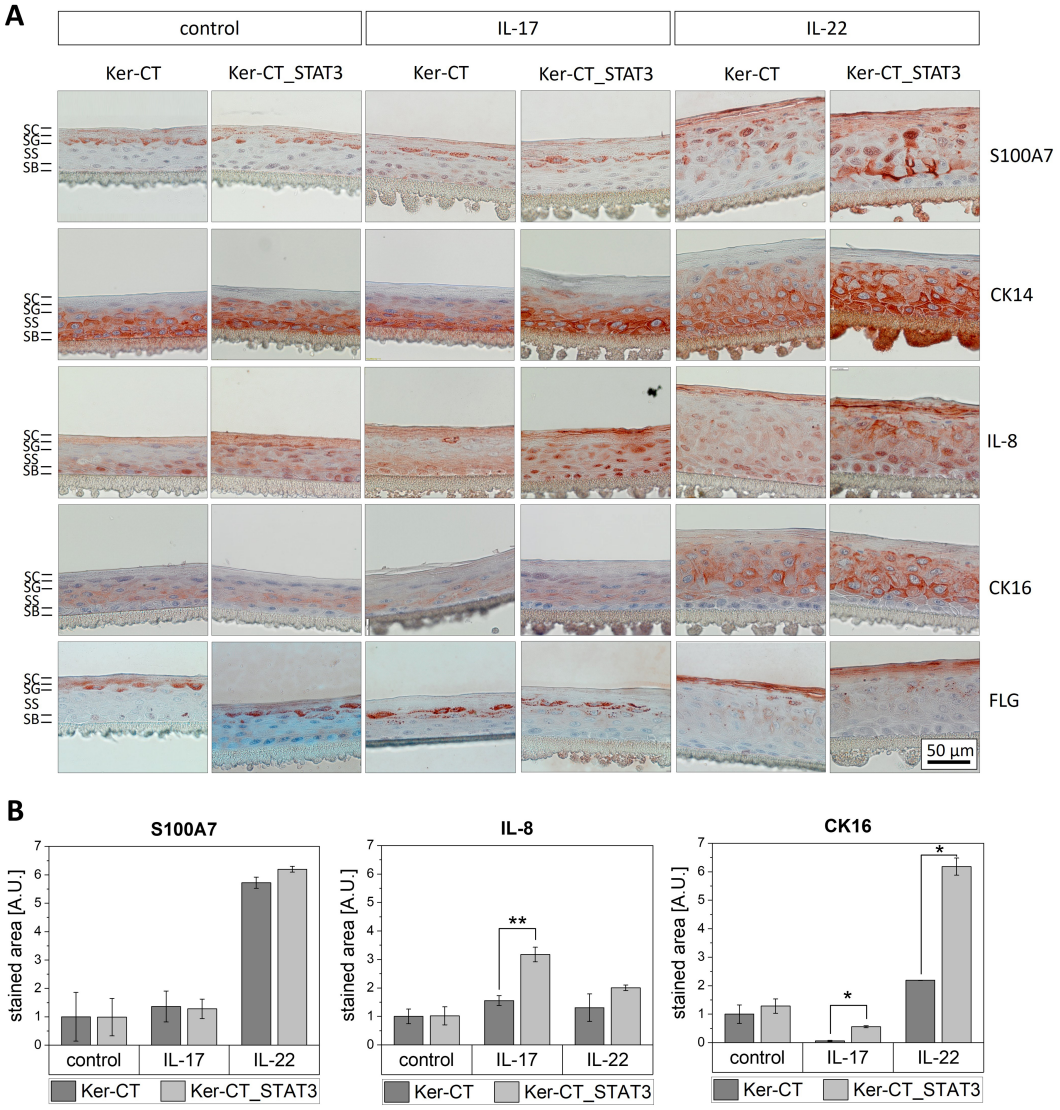
Stimulation of STAT3 overexpressing epidermis models with cytokines. Epidermis models were established from Ker-CT or Ker-CT_STAT3 keratinocytes and treated with IL-17 (20 ng/mL) or IL-22 (20 ng/mL) for 6 days during airlift culture. **(A)** Immunohistochemical staining of the differentiation marker cytokeratin 14 (CK14), fillagrin (FLG) and the psoriasis marker S100A7, IL-8 and cytokeratin 16 (CK16). Bar = 50 μm. The different epidermal layers *stratum basale* (SB), *stratum spinosum* (SS), *stratum granulosum* (SG) and *stratum corneum* (SC) are indicated for the control condition of the normal epidermis models. **(B)** Quantification of the stained area in all epidermal layers in the models for the indicated psoriasis markers. *p*-value (*p < 0.05, **p < 0.01) was determined with student’s *t*-test.

Next, we set up epidermis models from the Ker-CT_STAT3 keratinocytes. We explored how the epidermis models with the STAT3 overexpressing keratinocytes responded to an inflammatory environment induced by IL-17 and IL-22 and compared it to epidermal equivalents of the wild-type cell line (Ker-CT) (Figure 2A). We analyzed the effects on epidermal stratification with immunohistochemical staining for skin differentiation markers. The localization and the intensity of the respective marker was determined by representative microscopic images. Filaggrin, a late differentiation marker, was detected in non-treated models of both STAT3 overexpressing and normal epidermis models in the keratohyalin granules in the *stratum granulosum* and weakly in the *stratum corneum*. Upon treatment with IL-22, filaggrin was only expressed in the *stratum corneum* whereas IL-17 reduced filaggrin expression to the *stratum granulosum*. Interestingly, the decrease in filaggrin expression in presence of IL-17 was stronger in STAT3 overexpressing models than in equivalents with the base keratinocyte cell line. Cytokeratin 14 (CK14) was detected in non-treated epidermis models in the *stratum basale* and declined with increasing differentiation. IL-22 treatment enhanced the CK14 expression in upper epidermal layers, this effect was more pronounced in STAT3 overexpressing epidermis models. We also investigated the effect of the cytokines on the expression of psoriasis markers by immunohistochemical staining. Cytokeratin 16 (CK16) and S100A7, are common psoriasis marker also used in the clinic (39). The microscopic images showed that in non-treated epidermis equivalents CK16 and S100A7 expression was low and restricted to the *stratum spinosum* and the *stratum granulosum*, respectively. IL-22 treatment strongly upregulated the expression of both markers in all epidermal layers except for the *stratum basale*. The quantification of the CK16 staining intensity over all epidermal layers in cross-sections of the models indicated that the effect of IL-22 treatment was significant enhanced in the STAT3 overexpressing epidermis models compared to normal epidermis models (Figure 2B). Finally, we analyzed IL-8 expression, a cytokine produced by keratinocytes. IL-8 was significantly stronger elevated in STAT3 overexpressing epidermis models upon IL-17 treatment than in epidermis models of the base cell line (Figure 2B). The representative microscopic images showed that the expression pattern of the cytokine varied in different epidermal layers (Figure 2A). While IL-8 was located in the basal layers in the nucleus, it was detected in upper layers in the cytosol. In contrast to the other psoriasis markers, the strongest effect on IL-8 expression in the models was observed upon IL-17, not IL-22 treatment (Figure 2B).

### 3.3 Analysis of sensitivity to cytokine treatment of STAT3 overexpressing epidermis equivalents

**Figure 3:**
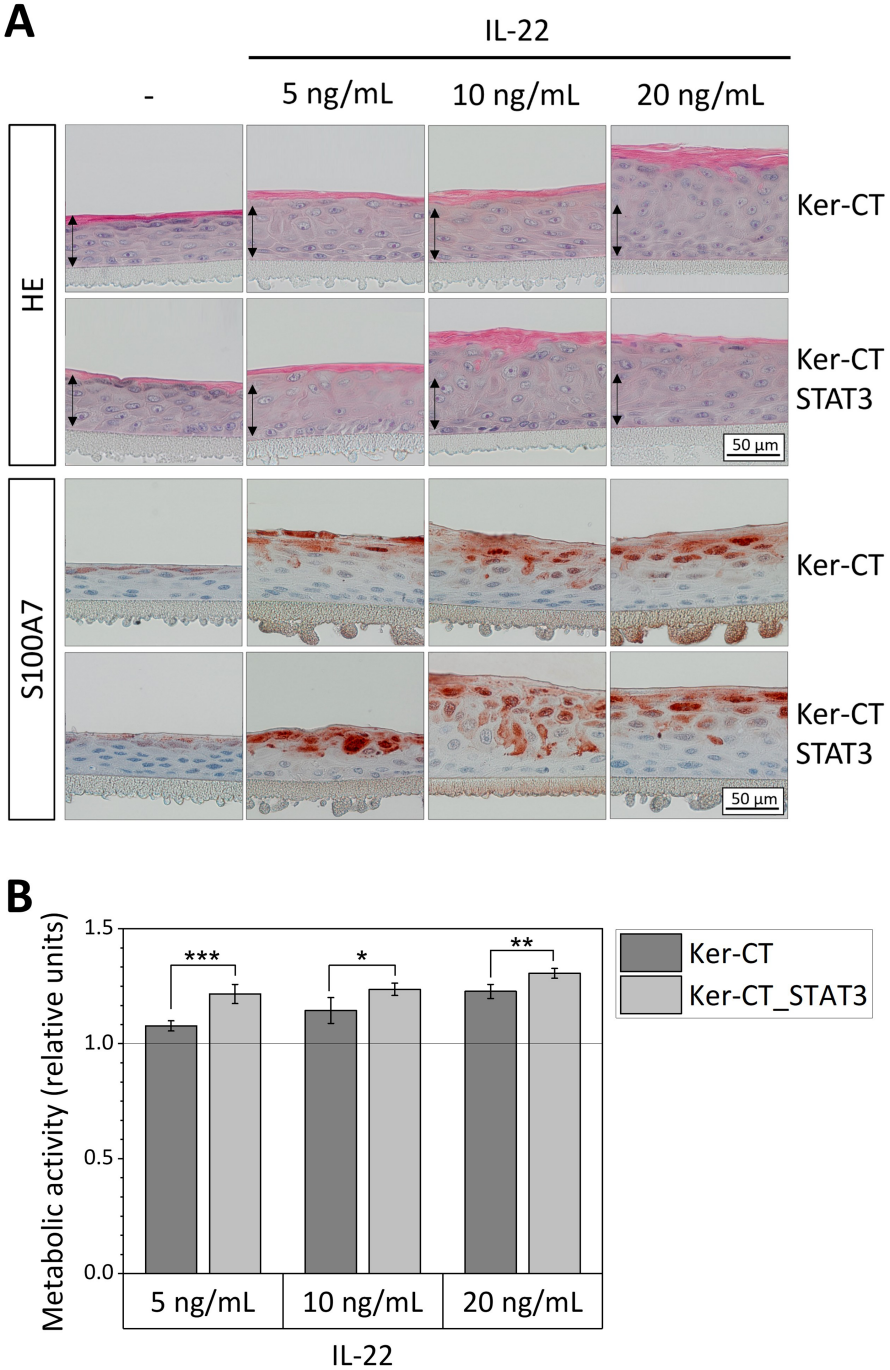
Dose–response analysis of IL-22 in STAT3 overexpressing and normal epidermis equivalents. Epidermis models were established from wild type (Ker-CT) or STAT3-eGFP keratinocytes (Ker-CT_STAT3) and stimulated for 6 days with 5, 10, or 20 ng/mL IL-22. **(A)** The epidermis models were HE stained and immunohistochemically analyzed for the expression of S100A7. The arrows indicate the height of the untreated normal (Ker-CT) epidermis model. Bar = 50 μm. **(B)** Metabolic activity of normal and STAT3 epidermis models upon treatment with different concentration of IL-22 was determined with an alamarBlue® Assay. Fluorescence intensity was normalized to the control condition of the respective cell line. *p*-value (*p < 0.05, **p < 0.01, ***p < 0.001) was determined with student’s *t* test.

IL-22 exhibited a strong effect on the skin equivalents for all analyzed marker proteins with the STAT3 overexpressing models responding with a more pronounced expression compared to normal equivalents. To study the sensitivity of normal and STAT3 overexpressing epidermis equivalents for cytokines in more detail, the equivalents were treated with different concentrations of IL-22 (Figure 3). Both, the normal and the STAT3 overexpressing epidermis equivalents responded to the lowest concentration of IL-22 employed in the experiment. Histological staining with hematoxylin/eosin showed a thickening of the epidermis (acanthosis), the absence of a *stratum granulosum* and a partially impaired cornification in epidermal equivalents treated with IL-22. This effect was more pronounced with increasing concentration of IL-22. The STAT3 overexpressing equivalents exhibited in immunohistochemical staining a higher S100A7 expression at the lowest dose (5 ng/mL) and the medium dose (10 ng/mL) of IL-22, if compared to the epidermis generated from unmodified Ker-CT cells. Thus, IL-22 at low doses had a stronger impact on the differentiation in STAT3 overexpressing than in normal epidermis models. We furthermore analyzed the metabolic activity of keratinocytes using a cell viability assay as described in the methods section to quantify the effects (Figure 3B). The metabolic activity of the cells in both epidermis models increased with escalating concentrations of IL-22. At all concentrations of IL-22, the metabolic activity in the STAT3 overexpressing epidermis models was significantly higher than the metabolic activity of the wild-type epidermis equivalents.

### 3.4 Integration of immune cells in STAT3 in vitro models

**Figure 4:**
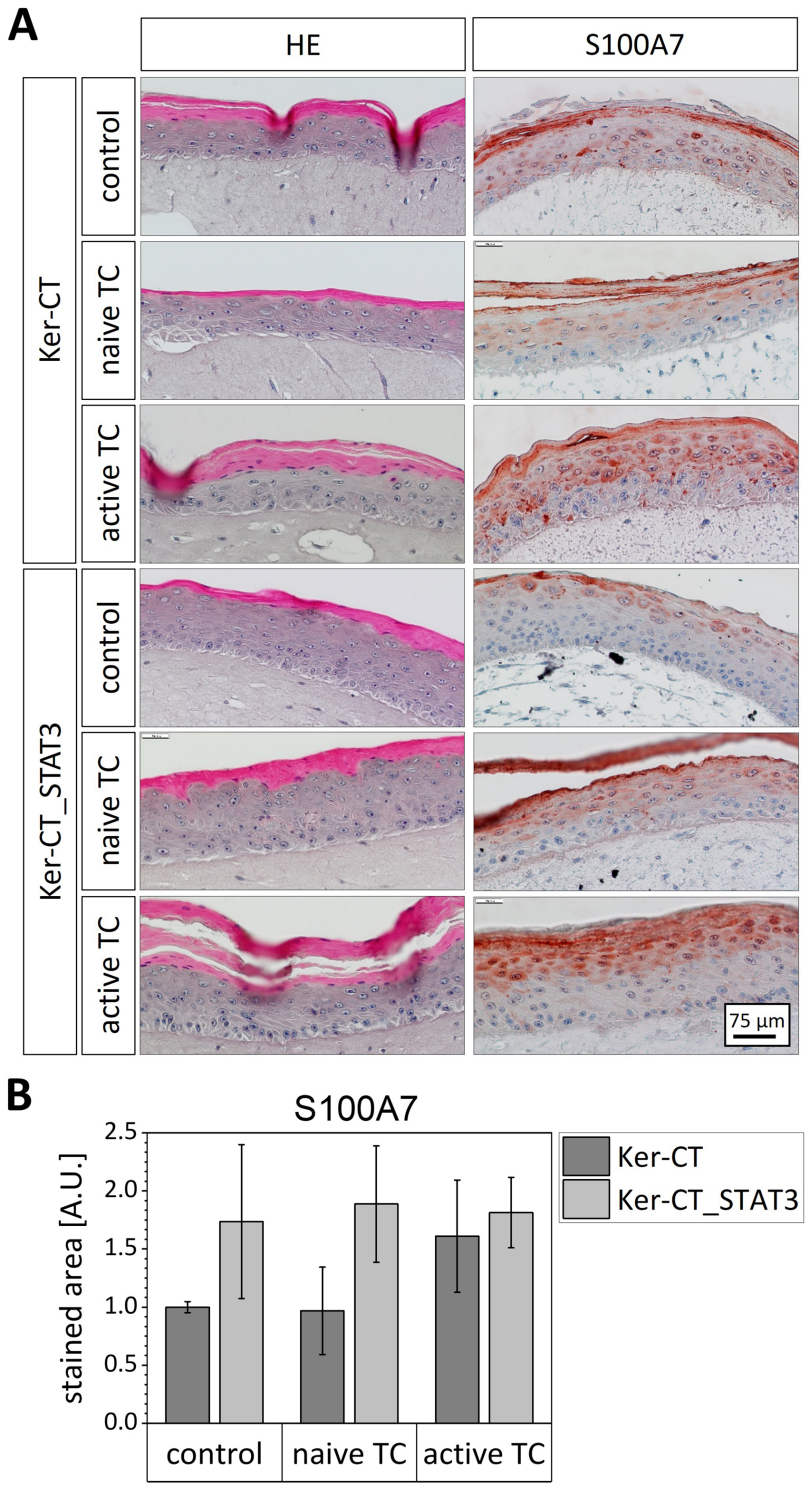
Integration of CD4 ^+^ T cells in full-thickness skin models set up from normal and STAT3 overexpressing keratinocytes. CD4 ^+^ T cells were isolated from fresh blood and used directly or stimulated in vitro with CD3/CD28 beads. T cells without stimulation (naïve TC) and T cells after stimulation (active TC) were integrated in the models beneath the dermal compartment for 3 days. **(A)** Skin models were HE stained and analyzed immunohistochemically for S100A7 expression. **(B)** The S100A7 staining intensity over all epidermal layers in the cross-sections of the models was quantified and normalized to the control skin models set-up from the wild-type cell line Ker-CT.

To mimic the in vivo situation more closely, we generated full-thickness skin models including both an epidermal part set-up from wild-type or STAT3 overexpressing keratinocytes and a dermal part containing fibroblasts. The psoriasis-relevant cytokines IL-17 and IL-22 have been shown to be produced and secreted in vivo by Th17 and Th22 cells (40, 41). Since Th17 and Th22 are both T helper cells belonging to the CD4^+^ T lymphocytes, we integrated CD4^+^ T cells into both skin models striving to replace the exogenous addition of cytokines. CD4^+^ T cells were isolated from blood of healthy donors and either used without stimulation or stimulated in vitro with CD3/CD28 beads. The use of CD3/CD28 beads for expansion and activation of CD4^+^ T cells is a well-described method (33, 42–44). Integration of the stimulated CD4^+^ T cells resulted in significantly greater cornification of the models and, at the same time, incomplete differentiation, visible by the retention of the cell nuclei in the *stratum corneum* of the epidermis (Figure 4A). The skin model with normal keratinocytes showed a strong expression of the psoriasis marker S100A7 if activated CD4^+^ T cells were integrated (Figure 4B). In contrast, the supplementation of this model with naïve T cells did not affect the S100A7 expression. Strikingly, STAT3 overexpressing skin models showed upon all conditions higher S100A7 expression than normal skin models. In the control condition, the S100A7 level in the STAT3 overexpressing skin models was already as high as in normal skin models after addition of activated T cells (Figure 4B). Noteworthy, the presence of naïve or activated T cells did not further increase S100A7 expression in STAT3 overexpressing skin models.

## 4 Discussion

There are numerous in vitro models and more than 40 different mouse models for psoriasis that have contributed to our current knowledge of the disease (19, 34). For years, STAT3-variants have been used in mouse models (e.g., K5.STAT3C mouse model) to mimic psoriatic conditions. Mice experiments showed that constitutive active STAT3 in keratinocytes induced psoriasis-like lesions and that especially the STAT3 signaling in keratinocytes and not in immune cells is essential for the pathogenesis (45). However, the transferability from mice to human skin is limited. Mice have a thinner dermis and epidermis, they have a higher keratinocyte turnover rate and the immune system differs in many aspects between mice and humans (19). Most of our knowledge about the role of STAT3 in psoriatic keratinocytes is the result of mouse experiments, some of it has been confirmed with samples from psoriatic patients (12, 14, 45). Yet, there has been no human based in vitro model for the role of STAT3 in keratinocytes. We addressed this lack and established a monoclonal STAT3 overexpressing human keratinocyte cell line. Endogenous STAT3 is constitutively expressed in cells at a low basal level and transcription is rapidly upregulated upon activation (46). The exogenous STAT3-eGFP we integrated in the genome is under control of a CMV promoter which contributes to a constitutive expression of STAT3. It does not enable regulation of STAT3-eGFP on a transcriptional level. However, in opposite to the K5.STAT3C mouse, which has a constitutive active STAT3, the activity of STAT3-eGFP in our construct is regulated by phosphorylation. The overexpressed STAT3-eGFP protein was phosphorylated in addition to the endogenous STAT3 in response to treatment with cytokines leading to a higher overall amount of active STAT3 in the keratinocyte cell line (Figure 1). We confirmed that the C-terminal eGFP tag did not affect the proteins phosphorylation, as shown by others (38), making the novel Ker-CT_STAT3 cells well suited for studying STAT3 hyperactivation in psoriasis.

### 4.1 STAT3 overexpressing keratinocytes respond with higher sensitivity to cytokine stimuli

Next, to genetic components, psoriasis pathology is marked by inflammatory feedforward loops between immune cells and keratinocytes. Especially T helper cells, such as Th17 and Th22 which secrete the cytokines IL-17 and IL-22 play an essential role in psoriasis. It was shown by our group and others that the psoriasis-associated cytokines IL-17 and IL-22 are suited to mimic psoriatic characteristics in 3D in vitro models (25, 27, 28, 47, 48). To test the STAT3 overexpressing keratinocytes in a psoriasis-like setting, epidermis models were set up from Ker-CT_STAT3 cells and treated with cytokines. The stimulation with cytokines IL-17 and IL-22 affected the epidermal stratification and induced expression of psoriasis protein markers in the epidermis models. The observed effects were significantly stronger in STAT3 overexpressing epidermis equivalents compared to epidermis models set up from the wild-type cell line Ker-CT (Figure 2). Consequently, we were (1) able to mimic psoriatic characteristics by stimulation of our epidermis models with cytokines and (2) STAT3 overexpression in keratinocytes further enhanced the impact of the cytokines, possibly by rendering the cells more sensitive to the pro-inflammatory environment. This was investigated more detailed with an escalating dose of IL-22. Epidermis models based on Ker-CT_STAT3 responded to IL-22 with stronger expression of the psoriasis marker S100A7, with more pronounced acanthosis and with an increased metabolic activity compared to the wild-type cell line Ker-CT (Figure 3). It is well established that IL-22 promotes keratinocytes proliferation and inhibits epidermal differentiation (49, 50). This was shown previously by others in 2D keratinocyte cultures, in 3D skin models as well as in in vivo mouse models. The histological staining showing strong acanthosis and the loss of the *stratum granulosum* in the epidermis models upon IL-22 treatment are in agreement with these previous results. To quantify the differences in the response of epidermis models set up from Ker-CT and Ker-CT_STAT3 to IL-22, we additionally measured the metabolic activity. STAT3 overexpressing epidermis equivalents exhibited for all concentrations of IL-22 a significantly higher metabolic activity indicating a higher number of vital keratinocytes which is the result of an increased keratinocyte proliferation and lower degree of keratinocyte differentiation. Which of these options is most prevalent in the Ker-CT_STAT3 model might reveal additional details in psoriasis development and will be addressed in further studies. Hyperproliferation and impaired differentiation are both psoriatic hallmarks (4), which consequently justifies the metabolic activity as read-out of psoriasis related effects. Thus, we showed by immunohistochemical staining and quantification of the metabolic activity that the epidermis models established from STAT3 overexpressing keratinocytes responded with a higher sensitivity to psoriasis associated cytokines. The stimulation of in vitro generated epidermal models with cytokines to mimic psoriatic characteristics is a well-known approach (25–31) which we could show in our unmodified epidermal models as well. In addition to other models, we have added a defined psoriasis predisposing factor to the model, the overexpression of STAT3. This allowed us to directly monitor the effect of STAT3 overexpression on the manifestation of psoriatic phenotypes in the model. However, other predisposing factors for psoriasis are not taken into account. Therefore, the STAT3 overexpressing keratinocytes in our model mimic only one aspect of the genetic predisposition of the disease. Nevertheless, the results in this work support that the STAT3 in vitro model mimics an intrinsic tendency towards a psoriatic phenotype that requires less external stimulation by cytokines to develop psoriatic characteristics. The combined analysis of microscopic images and marker intensity for the immunohistochemical staining of the epidermis models provides information on both the localization and the level of the protein marker expression. An exact quantification of the marker in the individual epidermal layers was not part of this study. Thus, we are aware that we might be missing some information that could be gained by a more differentiated quantification of the respective markers in future studies.

### 4.2 Ker-CT_STAT3 mimic psoriatic characteristics in a full-thickness skin model with immune cells

Compared to 2D monolayer culture, the 3D epidermis equivalents are highly physiological as they exhibit an epidermal stratification very similar to in vivo skin tissue (51). However, to generate a psoriasis model that mimics in vivo skin and especially skin immunity more closely, we integrated the STAT3 overexpressing keratinocytes in our established immune-cell supplemented full-thickness skin model (35). With naïve or activated T cells integrated beneath the dermis in a collagen layer, the model contains the main cellular players of psoriasis and enables investigation of the interplay between immune cells of different activation status, keratinocytes and fibroblasts. Instead of artificial supplementation of the medium with cytokines, the in vitro activated CD4^+^ T cells secrete naturally a pro-inflammatory cocktail of cytokines (23). In skin models with wild-type keratinocytes, the integration of activated CD4^+^ T cells led to similar psoriatic changes as the supplementation of the cell culture medium with cytokines, such as increased S100A7 expression and impaired epidermal stratification. Psoriatic effects were not observed if naïve T cells were integrated in the unmodified skin models (Figure 4). This indicates that the cytokine level secreted by naïve CD4^+^ T cells is not sufficient to activate a psoriasis-like phenotype and reflects the in vivo situation in which dermal cell and skin-resident T cells also co-exist in an inactivated status without inflammatory response (52). It also shows that the observed inflammatory reaction upon integration of activated T cells in the normal skin models is not an unspecific result of an allogenic reaction between the cells from different donors.

The STAT3 overexpressing skin models showed in the control condition a clearly higher S100A7 expression level than the wild-type skin models. Consequently, STAT3 overexpression by itself was sufficient to induce psoriatic alterations in the skin model. Supplementation of the STAT3 overexpressing model with activated or naïve T cells, did not significantly increase S100A7 levels, however, activated T cells in contrast to naïve T cells induced strong parakeratosis and hyperkeratosis, both hallmarks of psoriatic skin (Figure 4). The STAT3 overexpressing model thus reflects very closely the in vivo situation. In psoriasis patients, genetic risk factors play a substantial role in the development of the disease (53). This is mimicked in our model by STAT3 overexpression in keratinocytes. Studies that compare healthy, non-lesional and lesional skin in psoriasis patients, demonstrated that the non-lesional skin represents an intermediate state between healthy and lesional skin (54–56). Non-Lesional skin shows protein markers of lesional skin and exhibits higher sensitivity to inflammatory stimuli and hyperproliferation (39, 55). In our STAT3 overexpressing model, we observed this pre-psoriatic like phenotype of non-lesional skin. STAT3 overexpressing skin models without psoriatic stimuli resembled histologically normal skin but expressed abnormal high levels of the psoriasis marker S100A7 (Figure 4). We are not aware of any other in vitro model that was able to show this intermediate phenotype, and relate it to a single psoriasis-predisposing genetic alteration.

Psoriasis is accompanied by epidermal barrier dysfunction. We assumed that cytokine treatment and the presence of T cells also affected the skin barrier function as both inflammatory stimuli had strong impact on the morphology and expression of protein and differentiation markers in skin equivalents. Indeed, preliminary experiments, measuring the transepithelial electrical resistance (TEER) (data not shown) indicate a significantly compromised skin barrier upon IL-22 treatment in wild-type and STAT3 epidermis models. Further, the epidermal equivalents set up from STAT3 overexpressing keratinocytes also exhibited a compromised skin barrier at an intermediate level in the untreated condition although morphologically the STAT3 epidermis models resembled the wild-type models (Figures 2, 3). Thus, we assume that our STAT3 overexpressing model reflects not only psoriatic skin in terms of protein marker expression and histological hallmarks but also regarding the impaired skin barrier. Detailed studies to verify this are on the way.

It is highly interesting, that STAT3 overexpression in keratinocytes was sufficient to induce psoriasis marker expression in full-thickness skin models but not in epidermis models. This indicates that fibroblasts, which are only present in full-thickness skin models, contribute to inflammatory responses in the STAT3 overexpressing model as we have already demonstrated in a skin model for a *C. albicans* infection (36). This also shows the advantages of setting up in vitro models with increasing complexity in order to further understand the contributions of the individual cell types to the disease phenotype. Clarification of the exact role of fibroblasts and the interaction of the different skin cell types for the observed psoriatic inflammation in the model will be a focus of future studies.

### 4.3 Novel STAT3 psoriasis model is a versatile tool for preclinical research and an alternative to animal testing

3D in vitro skin models are well established for skin research, however not for inflammatory skin diseases such as psoriasis. It remains a major challenge to model the complex interaction between keratinocytes, fibroblasts and immune cells in the pathogenesis of psoriasis (6, 57). Further, most of the current 3D in vitro models are based on primary cells, which are isolated from leftover tissue from surgeries. The high inter-donor variability prevents a standardization of the models. Our psoriasis model is highly reproducible as it is based on STAT3 overexpressing immortalized primary keratinocytes. Starting from the STAT3 keratinocytes, additional 3D models can be established in a modular way. STAT3 overexpressing epidermis equivalents are a reproducible model, well suited for standardization and automatization and still the model exhibits many psoriatic hallmarks and an increased inflammatory response to psoriasis-associated cytokines in comparison to models set up from the wild-type keratinocyte cell line. We also generated an immunocompetent STAT3 overexpressing full-thickness skin model which is equipped with immune cells and a dermal layer with fibroblasts and, thus, providing higher similarity to in vivo skin. To our knowledge, this STAT3 overexpressing skin model is the first in vitro full-thickness psoriasis model which is set-up from immortalized primary keratinocytes – genetically modified - and fibroblasts, supplemented with immune cells. The high reproducibility of the psoriasis model makes it suitable for drug development. The in vitro psoriasis models shown here were cultivated for up to 2 weeks under airlift condition. A longer cultivation period, which is of high interest for pharmacological studies, has to be tested in future experiments. 3D skin equivalents for other purposes, without immune cells, have been cultivated for up to 4 weeks in our laboratory. An extended culture duration could enable the investigation of longer-term effects of chronic skin inflammation on the skin barrier and psoriasis marker expression. Histological, immunohistochemical and TEER analysis as well as defined addition of interfering compounds at several time points within the lifetime of the models could lead to new insights into the sequence of pathogenic events during progression of psoriasis in a human-based model.

The focus on key cellular players in our in vitro model enables the specific analysis of cellular interactions in an in-vivo like 3D setting. However, the lack of other psoriasis relevant immune cell populations represents a limitation of the model. Further, it has to be considered that with the current in vitro models, we cannot study distant organ interactions. So, for the analysis of common psoriasis comorbidities like psoriatic arthritis or Crohn’s disease we still rely on in vivo models.

None of the current in vivo or in vitro psoriasis models matches all characteristics of the disease. Test models have to be picked depending on the research question. With our novel established human 3D in vitro STAT3 psoriasis model, we complement the current selection with an in vitro model that is especially suited for medium throughput screening approaches due to the high reproducibility, that offers a human based alternative to the current STAT3 mouse models and enables further validation of the STAT3 signaling pathway in keratinocytes as drug target. In future studies this model can be expanded in a modular way to add additional characteristics of psoriasis, including additional genetic alterations, additional types of immune cells or skin microbiota. To mimic the psoriasis associated microbial dysbiosis and explore effects of probiotics on psoriasis, skin resident fungi or bacteria could be integrated on the *stratum corneum* of the STAT3 overexpressing skin equivalents according to the protocol for our previously established 3D skin infection model (35).

## 5 Conclusion

Mice experiments showed that constitutive active STAT3 in keratinocytes induced psoriasis-like lesions and that especially the STAT3 signaling in keratinocytes and not in immune cells is essential for the pathogenesis (45). In our work, we confirmed that STAT3 signaling pathway in human keratinocytes mediates psoriatic characteristics in the skin. We mimicked genetic predisposition of psoriasis patients with STAT3 overexpression in Ker-CT keratinocytes and reproduced a pre-psoriatic like phenotype of non-lesional skin. This pre-psoriatic like phenotype was characterized by higher sensitivity to cytokine treatment and an elevated basal expression of the psoriasis marker S100A7 in STAT3 overexpressing full-thickness skin models. Upon a pro-inflammatory stimulus, STAT3 overexpressing skin equivalents developed enhanced psoriatic characteristics compared to normal skin models. This corresponds to the in vivo situation in psoriasis patients, where formation of psoriatic lesions is also induced by an internal or external trigger in patients (58). Thus, our novel in vitro model mimics many psoriatic characteristics that can be attributed to STAT3 overexpression. As it is based on a cell line, its high reproducibility makes it well suited for drug screening approaches and validation of new therapeutics for the treatment of psoriasis.

## Data Availability

The original contributions presented in the study are included in the article/supplementary material, further inquiries can be directed to the corresponding author.
